# Comparative Study of Batch and Continuous Flow Reactors in Selective Hydrogenation of Functional Groups in Organic Compounds: What Is More Effective?

**DOI:** 10.3390/ijms241814136

**Published:** 2023-09-15

**Authors:** Marina V. Bukhtiyarova, Alexey L. Nuzhdin, Galina A. Bukhtiyarova

**Affiliations:** Boreskov Institute of Catalysis SB RAS, 630090 Novosibirsk, Russia; anuzhdin@catalysis.ru (A.L.N.); gab@catalysis.ru (G.A.B.)

**Keywords:** selective hydrogenation, batch reactor, continuous flow reactor, nitro group, carbonyl group, unsaturated carbon bonds

## Abstract

Many research papers describe selective hydrogenation of functional groups, such as nitro groups, carbonyl groups, or unsaturated carbon bonds to obtain fine chemicals or precursors of pharmaceuticals. Quite often, the catalyst performance is investigated in batch or continuous flow reactors without finding advantages and disadvantages of this or that regime. At the same time, the transition from batch processes to continuous flow occurs on the industrial scale. However, the batch process can be preferable for some reactions, in spite of its drawbacks. This review article aims to identify all publications that consider selective hydrogenation of functional groups in organic compounds, both in batch and continuous flow reactors, at the same reaction conditions that allow making conclusions about the benefits of one of the regimes in a particular case.

## 1. Introduction

**Figure 1:**
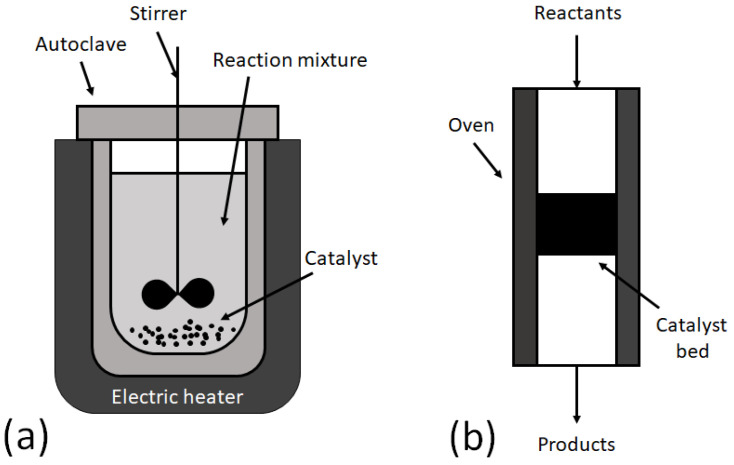
Schematic diagram of batch (**a**) and continuous flow (**b**) reactors.

Most reactions of petrochemical and commodity chemical industries are heterogeneous catalytic processes, which have been studied in detail. Historically, the manufacturing of fine chemicals has been developed with batch technologies (Figure 1a) [1,2]. Mostly, this approach remains the main production technique in industries due to the high profit with low cost and flexibility of batch units to make multiple products. At the present moment, the synthesis of fine chemicals in continuous flow mode (Figure 1b) is an interesting challenge for researchers all over the world. On the other hand, developing flow processes can be time consuming. 

The batch reactor is a transient reactor. An autoclave uploaded with a reaction mixture and catalyst is commonly used as a batch reactor to perform a reaction at high temperature and high pressure. The main characteristics of batch reactors are the following:

- the reaction is performed in the liquid phase;
- temperature control and vigorous stirring is needed to make sure that the temperature and composition are uniform in the whole volume of the reactor;
- concentrations of reactants and products are changed with the clock time, meaning that the longer the reaction time, the higher the product yield is;
- long synthesis can result in catalyst deactivation without knowing it has happened;
- catalyst deactivation is determined by reactivation of the catalyst and repeating the catalytic run with a washed catalyst;
- catalyst particles disturb the sampling procedure by possibly blocking the sampling port, if it is present in the autoclave;
- batch synthesis should be repeated several times to produce a high amount of the desired product.

A continuous flow reactor is a steady-state reactor, in which the reagents are fed constantly to the inlet of the reactor and move through the catalyst bed. After the reaction, the mixture of unreacted reagents and products flow at the outlet. The main characteristics of continuous flow reactors are the following:

- the reaction is performed in the gas phase;
- the composition of the gas at the outlet does not change with the clock time;
- precise control of the molar ratio of reactants is possible by controlling the flow rates of reactants;
- the change of residence time without changing the catalyst in the reactor;
- catalyst deactivation is determined by the long-term stability test with online measurement of the gas mixture;
- there is no need to start and stop the continuous process for the production of the target product in a high yield.

Authors [3,4] made an attempt to create a diagram that can help to choose between the batch and continuous flow mode to perform the catalytic reaction with appropriate parameters. The catalytic process is more convenient and reliable in the batch reactors if:

- there is an acceptable level with respect to yield, scale, and reaction time;
- the existing synthesis route fits the existing batch equipment;
- the immediate goal is optimization of discrete variables;
- there is low market growth (<1 kt/a);
- precipitate drives the reaction to completion.

On the other hand, continuous flow reactors are preferred if:

- one of the reagents is gas;
- the reaction is performed over a heterogeneous catalyst;
- there is high marker volume (>10 kt/a);
- products suffer under catalyst deactivation;
- heating accelerates the reaction.

Usually, researchers [5,6,7] postulate that performing reactions in flow has several advantages in comparison to processes carried out in batch reactors, such as better mixing of reagents, excellent interfacial mass and energy transfer properties, lower operational costs, suppression of byproduct formation through better control over reaction parameters, simplified scaling up, and improved process safety.

Reactants are introduced continuously to the reactor in such a way that only a limited quantity of them react at a given time, meaning well mixing by rapid diffusion in a small reaction space. 

A low ratio of reactor inner diameter to catalyst particle diameter is needed for heat management, i.e., sufficient heat removal to the reactor wall for highly exothermic reactions. Moreover, reactor material has an impact on the heat transfer efficiency and needs to be chosen for an exact purpose. It is postulated that dimensions of flow reactors can be smaller than that of batch reactors to obtain the same yield and selectivity [4]. Small reactors promote efficient heat exchange. It is worth noting that the energy costs are decreased by enhanced heat transfer. 

Due to the absence of headspace in flow reactors, there is no accumulation of unstable intermediates. Furthermore, side products, which can block active sites of the catalysts, are removed from the catalyst by the flow. The step of catalyst separation from products is avoided in the case of flow reactors. 

In general, scaling up a batch system is easier since batch reactors are mostly available on any required scale. It is only necessary to select the proper stirrer and heating jacket for a batch reactor of high dimensions. For the scale-up of flow reactor kinetic data, reactor modeling and heat and mass transfer information are needed for obtaining the safety process. In addition, clogging issues are greater in the case of flow reactors [4]. 

However, as it was mentioned in [3], these benefits are not always relevant to the topic of the paper. Quite often, researchers try to replace batch reactors with continuous flow reactors due to their better performance, while comparison of the catalytic activity of the same catalytic systems in both reactors is absent in the paper. Readers should believe that the investigated catalysts work better in the continuous flow mode. 

There is a lack of literature that has compared the catalytic performance in both batch and continuous flow modes. The aim of the present review is to reveal all publications that consider selective hydrogenation of different functional groups (nitro and carbonyl groups or unsaturated carbon bonds) in batch and continuous flow reactors and compare the performance (activity and selectivity) of the same catalytic systems.

## 2. Selective Hydrogenation of Functionalized Nitroarenes

Functionalized anilines are extensively used as intermediates in the agrochemical, pharmaceutical, and fine chemical industries [8,9]. They are synthesized through catalytic hydrogenation of a nitroaromatic precursor. It is challenging to hydrogenate nitro groups selectively in polyfunctional nitroarenes containing unsaturated functionalities, such as multiple carbon bonds and cyano and carbonyl groups, which can be also reduced.

### 2.1. Halogenated Nitroarenes

Selective catalytic hydrogenation of aromatic halonitroarenes is an important process to yield haloanilines, which can be used as intermediates for industrial products, such as dyes, perfumes, drugs, herbicides, and pesticides [10,11]. If halogen is present in addition to the nitro group in nitroarenes, hydrodehalogenation can occur that results in formation of nitrobenzene and aniline. 

Authors [12,13] carried out selective hydrogenation of p,o-chloronitrobenzene (p,o-CNB) to p,o-chloroaniline (p,o-CAN) over Pd- and Au-supported catalysts, both in batch and continuous flow reactors. Liquid-phase reactions (H2 pressure of 5–12 bar and temperature of 150 °C) were carried out in a commercial batch-stirred stainless steel reactor of 100 mL, while the gas-phase reaction occurred in a fixed-bed glass reactor (inner diameter of 15 mm) at atmospheric pressure and 220 °C. The reaction conditions were chosen to minimize heat and mass transfer limitations [14]. 

The first attempt to demonstrate the feasibility of selective hydrogenation of halogenated nitroarenes in continuous flow mode was conducted in [12]. The combination of Au with Mo_2_N was used as a catalyst, since Mo_2_N can chemisorb hydrogen, and Au is a selective metal to nitro group reduction. The synthesis of Au/Mo_2_N (0.25 wt.% of Au) was present in details in [12]. Later on, X. Wang et al. [13] considered Au/TiO_2_ (0.1 wt.%) as a catalyst for hydrogenation of ortho-chlorobenzene. This material was synthesized by the deposition–precipitation method described in [15]. 

The catalytic performance was investigated in the batch liquid phase over Pd/C [13] and the continuous flow gas phase over Au/Al_2_O_3_ as benchmarks [12]. Besides the target product, byproducts such as nitrobenzene and aniline can be obtained during hydrogenation of chloronitrobenzene over Pt-, Rh-, Ru-, Ni-, and Cu-supported catalysts [16,17,18]. A comparative table of the catalytic behavior of all materials at different reaction conditions is present below (Table 1).

Performing reactions in the batch liquid phase is standard practice in the pharmaceutical sector. It was shown [13] that the main product of the reaction at 5 bar over Pd/C was ortho-CAN with a selectivity of 79% (Table 1). The full conversion was achieved in 1 h. An increase in H_2_ pressure led to an increase in the target product selectivity and a decrease in aniline selectivity (Table 1). Unwanted side reactions should be minimized; that was possible to achieve by using Au-containing catalysts. Complete conversion of o-CNB was achieved in 30 h over Au/TiO_2_ at hydrogen pressure in the range of 5–12 bar and a temperature of 150 °C. The Au/Mo_2_N showed complete conversion of p-CNB with 100% selectivity of p-chloroaniline after 27 h of experiment at 11 bar and 150 °C (Table 1). Catalytic activity was quantified by the initial reaction rate. The reaction rate obtained over Au/TiO_2_ was lower than that over Pd/C at any reaction conditions (Table 1) due to the lower ability of supported Au to adsorb H_2_ than that of Pd [13]. The higher H_2_ pressure led to a higher o-CAN yield. 

To overcome the safety concerns associated with pressurized H_2_, the continuous gas flow experiments were performed at atmospheric pressure, lowering energy requirements and minimizing safety hazards. The temperatures of 220 and 150 °C were used for selective hydrogenation of the nitro group in chloronitrobenzenes over Au/Mo_2_N and Au/TiO_2_, respectively (Table 1). Setting a higher temperature in the case of Au/Mo_2_N was not explained by F. Cárdenas-Lizana et al. [12]. However, performing the reaction in the continuous mode at the same reaction temperature as that in the batch mode would allow comparing obtained results more precisely.

The catalytic activity of the Au/Mo_2_N, Au/Al_2_O_3_ (benchmark), and Pd/Mo_2_N catalysts was compared in continuous flow hydrogenation [12]. Pd/Mo_2_N was selected due to enhanced activity in nitro group reduction [19]. The Au/Al_2_O_3_ catalyst showed lower catalytic activity in comparison to Au/Mo_2_N, probably since there was no surface cooperative effect between Au and Al_2_O_3_, which could lead to enhanced hydrogen uptake in this system (Table 1). However, selectivity to the target product was 100% for both Au-containing catalysts. Pd/Mo_2_N revealed the higher hydrogenation rate in comparison to Au/Mo_2_N, meaning the higher hydrogen activity [20], while the selectivity to p-CAN dropped significantly to ~55% due to C-Cl hydrogenolysis with formation of aniline and nitrobenzene (Table 1). Thus, neither Pd/C nor Pd/Mo_2_N catalysts are applicable materials in industrial processes for selective hydrogenation of para-chloronitrobenzene without downstream separation of the target product since the additional process of C-Cl hydrogenolysis occurs. 

It should be noted that running a liquid-phase reaction in the batch reactor requires three recharges per week for stable catalyst work, which consists of the following steps: reactor loading, catalyst activation, temperature/pressure stabilization, extraction of product/recharge with reactant, catalyst filtration/washing/reloading. Ten hours of work are needed to perform all these steps. The continuous flow operation mode can run without the two last steps, extraction of product/recharge with reactant and catalyst filtration/washing/reloading, which reduces the working time to 3 h. Productivities, which are influenced by several factors, were calculated on the basis of 7 working days for both operation modes over Au/TiO_2_ [6]. The move from batch to continuous mode decreases the working time with the increase in productivity to target o-chloroaniline from 5·10^3^ to 86·10^3^ kg_o-CAN_·kg_Au_^−1^·year^−1^.

Authors [21,22] investigated the catalytic reduction of 1-iodo-4-nitrobenzene (INB) to 4-iodoaniline (IAN) as a model reaction to assess catalytic activity and chemoselectivity since the iodine substituent among halogens is most easily cleaved from the nitroarene molecule. Hydrogenation of INB can occur, with the formation of aniline and nitrobenzene as main byproducts. Therefore, a catalyst should be active enough for the production of IAN in a high yield with the minimized hydro-deiodination process. The reaction was performed in batch and continuous flow modes [21,22]. 

The studies were performed over low-cost Co-containing catalysts: Co Raney (15 mol% Co) [22] and polyurea microencapsulated Co catalyst (15 mol.% Co, Co EnCatTM) [21]. The effect of the substrate concentration was investigated for both catalysts in the batch reactor. However, the conditions of catalytic reactions were different (Table 2). 

In spite of different reaction conditions for both catalysts, the same conclusions about catalytic performance can be made. The increase in the substrate concentration resulted in a decrease in the 4-iodoanaline yield, as well as substrate conversion, while dehalogenation to aniline remained at the same level for both catalysts. This was a significant advantage for batch process since high pressure and temperature limits fast separation of the catalyst from reaction media. 

Scale-up experiments in batch process were performed over a Co Raney catalyst (15 mol % Co) at the following reaction conditions: 110 °C, 20 bar of H_2_, 10 mL of THF:H_2_O (95:5), substrate concentration of 0.05 mol·L^−1^ [22]. Scaling up was performed in two steps: from 10 to 200 mL reaction volume (300 mL reactor) and from 200 to 1000 mL (1.8 L reactor). Heating was slower for the higher reaction volume (200 mL) that resulted in increasing reaction time to obtain full conversion of INB and maximum product yield (225 min vs. 50 min). P. Loos et al. [22] postulated that decreased catalyst performance can be caused by a hydrogen mass transfer limitation in a larger reactor volume. The 4-iodoanaline yield increased with the reaction time and reached around 80% in the 200 mL batch reactor after 450 min of experiment. For the scale-up to the reaction volume of 1000 mL, the P. Loos et al. used a multi-purpose high-volume stainless steel autoclave (1.8 L), which was used with a variety of noble-based catalysts, whose residuals affect the catalytic performance of Co Raney. Full conversion of 1-iodo-4-nitrobenzene was achieved in 225 min, as was that for the reaction volume of 200 mL. However, in reaching full conversion of 1-iodo-4-nitrobenzene, the yield of 4-iodoanaline started to decrease, meaning that its stronger dehalogenation to aniline occurred. P. Loos et al. [22] claimed that this phenomenon can be caused by residues of noble metals on the reactor walls. Therefore, a high-volume reactor specially made for using Co catalysts in the batch process is needed. 

Continuous experiments were carried out using a customized Ehrfeld modular cartridge microreactor 240 (5 mL). An increase in the IAN yield from 80 to 90% was obtained over Raney Co with the H_2_ pressure increase to 85 bar and the temperature decrease to 80 °C in comparison to the batch experiment (Table 2). The reason for changing the reaction conditions in such a harsh manner was not mentioned in the paper [22]. 

H. Alex et al. [21] studied the influence of temperature, substrate concentration (solvent THF:H_2_O = 95:5), and hydrogen pressure on catalytic activity of a Co EnCatTM catalyst in selective hydrogenation of INB in the continuous flow mode. The optimal reaction temperature of 100 °C, which allowed obtaining 88% of the IAN yield and 2% of the aniline yield, was found. The decrease in the reaction temperature led to a reduction in the IAN yield, while its increase caused the formation of a higher aniline amount that should be avoided. An increase in the substrate concentration from 0.05 to 0.2 M does not affect the 4-iodoaniline yield in continuous flow experiments, which achieved ~89%, which is higher than the maximum yield of 72% for the batch experiment. At the same time, aniline formation was only 1% at full substrate conversion, which is preferable in comparison to the value obtained in the batch experiment (4%). The effect of the pressure over the Co EnCatTM catalyst was studying varying H_2_ pressure in the range of 25–85 bar at 100 °C. The 4-iodoaniline yield remained on the same level of 87–91% independence of the pressure changed. However, the higher pressure of 50–85 bar decreased the dehalogenation reaction in comparison to the pressure of 25 bar (aniline yield was 1 and 4%, respectively). Thus, the Co EnCatTM catalyst can tolerate fluctuation in the gas/flow rate over a relatively wide range of reaction parameters without significant change in the product composition. The productivity of 0.93 mmol 4-iodoaniline·h^−1^·mmol_Co_^−1^ for the Co EnCatTM catalyst was much higher than the 0.08 mmol 4-iodoaniline·h^−1^·mmol_Co_^−1^ for the Co Raney catalyst [21,22].

It was found that different Co catalysts are promising catalysts in selective hydrogenation of iodonitrobenzenes, both in batch and flow operations. Performing reactions at the same conditions (85 bar of H_2_, 100 °C, and 0.05 M of substrate) in batch and continuous flow reactors, the higher 4-iodoaniline yield was obtained in the flow operation (~90% vs. ~70%) due to the ability to choose the optimum residence time. However, the reactor for batch experiments should be designed especially for the Co catalyst to exclude the effect of noble metals residues on selectivity towards the target product due to the formation of undesired aniline, which is in agreement with data obtained in [23]. E.O. Pentsak et al. showed that stir bars made of PTFE can be contaminated by Pd metal species during organic reactions performed in batch reactors over Pd-containing catalysts. It was shown that negligible amounts of Pd artificially added to stir bars promoted conversion of 1-bromo-4-nitrobenzene on the level of 21–79%. Thus, PTFE coatings inside the autoclave can be contaminated with Pd metal species that can have a negative effect on the target reaction.

### 2.2. Nitrophenols

Another reaction of selective hydrogenation of nitroarenes investigated both in batch and flow regimes is reduction of 4-nitrophenols (4-NP) [24,25], which are one of the stable organic pollutants for agricultural and industrial wastewater [26,27,28]. Hydrogenation of 4-NP to para-aminophenol, a precursor to various analgesic and antipyretic drugs [29], is a useful approach to overcome the abovementioned problems. Sodium borohydride is used as the reducing agent instead of hydrogen in this reaction [30,31]. In a typical process in the batch, the reaction was performed in a beaker by adding the measured amount of catalyst to reaction media prepared by mixing sodium borohydride (NaBH_4_) with 4-NP. The hydrogenation conditions are present in Table 3. 

The reaction process was monitored using a UV-vis spectrometer. Y. Li et al. [25] studied the catalytic activity of a porous carbon-encapsulated Cu_x_O/Cu catalyst derived from N-coordinated MOF, while A.K. Srivastava et al. [24] synthesized Ag nanoferns-decorated carbon fibers by the electrodeposition method at different voltages (5–10 V). 

In the batch reactor, ultrafast conversion of 4-NP was completed in 25 s for a polycrystalline Cu_x_O/Cu/NC catalyst (0.125 mg) [25], while 12–33 min were needed in the case of the cAg-NF catalysts, depending on the preparation procedure [24]. The reaction time was reduced with an increasing amount of Ag-NF in the catalyst. The highly efficient performance of Cu_x_O/Cu/NC was explained by the synergetic effect between Cu_x_O and Cu nanoparticles, which accelerates the electron transfer rate in the catalytic reaction. The reusability of Cu_x_O/Cu/NC (0.075 mg) in the batch reactor was tested without regeneration in six cycles. 4-NP conversion achieved 95% within 80 s in the sixth cycle. The reaction rate constants increased from 0.0682 to 0.0806 s^−1^ in the first three cycles, which can be related to the activation of the catalyst during the reaction [32]. X. Sun et al. [32] studied the stability of an N-doped Cu/Cu_x_O/C foam catalyst in 4-NP reduction, using NaBH_4_ as the reducing agent in a standard quartz cell at room temperature in seven cycles. The morphology of the catalysts was investigated by the SEM technique, which showed a decrease in the particle sizes in the first three cycles, meaning the activation process for the catalyst. In the next two cycles, the reaction rate constant dropped to 0.0401 s^−1^, which can be attributed to the presence of 4-NP and 4-aminophenol on the catalyst surface. These molecules hinder the adsorption of new 4-nitrophenol, resulting in a decrease in catalytic activity. 

The reusability of the cAg-NF catalysts was studied for the catalyst containing 0.96 mg of Ag-NF in fibers by repeating the experiment seven times [24]. After each experiment, the catalyst was removed, washed, dried, and added to a new batch of 4-nitrophenol. The complete conversion was obtained in 16 min for the first cycle, while it needed 40 min for the seventh cycle. Such a decrease in catalytic activity can be caused by weight loss of the original catalyst sample (14.2 wt.%) that is attributed to the loss of fibers detachment of the catalyst during repeated washing and the catalytic experiment.

To estimate the industrial applicability of both catalysts in industrial wastewater treatment, flow-through-type reactors were constructed. It should be noted that these reactors have special designs that will be discussed below. 

An online catalytic reactor-HPLC system was used in catalytic hydrogenation of 4-NP over the Cu_x_O/Cu/NC catalyst. The detailed scheme is presented in [25]. The detection of the 4-NP concentration in the reaction media (0.1 mM 4-NP and 100 equivalent NaBH_4_) was conducted by the direct injection method of the reaction solution to the catalytic reactor. The amount of catalyst (5 mg vs. 0.075–0.125 mg) used in the flow operation was higher than that placed into the batch reactor (Table 3). The long-term stability test, which is important for the prospective industrial use of the catalyst in continuous flow reactors, showed that the conversion (>97%) remained unchanged for 12 h of the experiment at 0.2 mL·min^−1^. The used catalyst revealed the same structure and morphology as fresh catalyst, meaning its high stability under the reaction conditions. Y. Li et al. [25] assumed that the flow regime helped to remove 4-aminophenol from the catalyst surface, which resulted in enhanced catalytic activity. 

For cAg-NF, two different continuous flow reactors were constructed in such a way that the flow of the reaction mixture is along or perpendicular to the axis of the catalyst. The detailed scheme can be found in [24]. Flow rates were varied in the range of 0.5–20 mL·min^−1^. The highest 4-nitrophenol conversion of 87% was obtained at the lowest flow rate of 0.5 mL·min^−1^. The further increase of flow rates led to a drop in the conversion to ~5% for both directions of flow because of the too short residence time. In the moderate flow rates, higher conversion was obtained for the reactor containing the cAg-NF fibers perpendicular to the flow due to the easy penetration of the liquid to the boundary layers of the dendritic structure of the nanoferns of cAg-NF. The lower efficiency for the cAg-NF fibers along the flow direction could be caused by low contact of the catalyst fibers with reaction solution. The long-term stability test was not performed for this reactor. There is no real comparison of the catalyst performance in batch and continuous flow reactors. A.K. Srivastava et al. [24] postulated that “the reactor design for the continuous flow model can be easily scaled up for effluent treatment”. However, there are some doubts about this statement. More active catalysts and more convenient setups should be found for selective hydrogenation of 4-nitrophenols.

## 3. Selective Hydrogenation of Carbonyl Groups in Acids and Aldehydes Containing Additional Double Bonds

### 3.1. Hydrogenation of Furfural or 5-Hydroxymethylfurfural

**Scheme 1:**
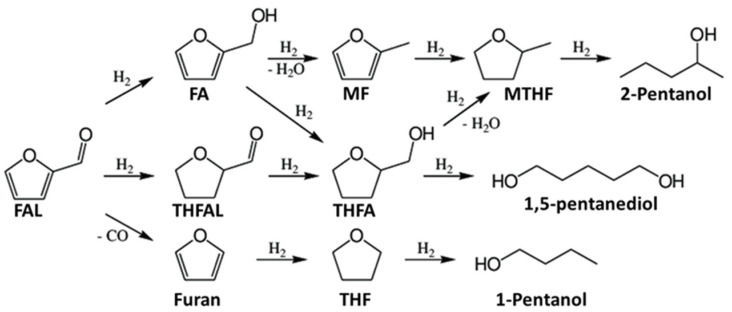
Furfural hydrogenation.

Non-edible plant-based feedstock can provide production of a wide range of high-value chemical compounds using catalytic processes. Among all chemicals derived from lignocellulosic biomass, furan molecules, such as furfural (FAL) and 5-hydroxymethylfurfural (HMF) [33], are the most attractive starting materials for the synthesis of different useful compounds [34,35,36]. The most significant process in FAL and HMF conversions is its hydrogenation. FAL is hydrogenated with the formation of furfuryl alcohol (FA), tetrahydrofurfural (THFAL), 2-methylfuran (MF), 2-methyltetrahydrofuran (MTHF), furan, tetrahydrofurfuryl alcohol (THFA), tetrahydrofuran (THF), and pentanediols (Scheme 1). 

FA is a valuable chemical for resins, flavors, fiberglass, varnish, and paints [37], while MF is an attractive molecule as an additive in gasoline fuels [38]. In the case of HMF, 2,5-bis(hydroxymethyl)furan is formed by its hydrogenation, followed by reduction of the furan ring to 2,5-bis(hydroxymethyl)tetrahydrofuran [39]. These products can be used in the synthesis of polyesters [34]. 

Copper–chromium alloy is used as a catalyst for hydrogenation of furfural to furfuryl alcohol for industrial application [40]. However, this process is environmentally dangerous due to the high toxicity of chromium, which can contaminate FA and, as a result, pharmaceutical products. It can be expected that Cu-containing catalysts promoted with other transition metals can be promising candidates in FAL hydrogenation. Researchers compared the catalyst performance in two operation modes (batch and continuous flow) over metal supported catalysts [37,41,42,43]. Comparison of the catalyst performance between batch and continuous flow modes was reported for two hydrogenation processes of furfural to furfuryl alcohol and 2-methylfuran.

One of the works [42] concerning comparison of the catalyst performance between batch and continuous flow reactors studied Cu-containing catalysts promoted with iron in furfural hydrogenation to furfuryl alcohol. It was shown earlier [44,45] that Cu-based materials can be active and selective in this reaction. The series of catalysts was synthesized by alloying the iron, aluminum, and copper nitrates at required ratios at 180 °C, with following calcination at 450 °C for one hour. The obtained catalysts are mixed oxides of the following compositions: Fe_82_Al_18_, Cu_5_Fe_78_Al_17_, Cu_10_Fe_74_Al_16_, Cu_20_Fe_66_Al_14_, Cu_30_Fe_57_Al_13_, Cu_40_Fe_49_Al_11_, Cu_50_Fe_41_Al_9_, and Cu_5_Al_95_, where the numbers are the concentration of the corresponding oxide in wt.%. Furfural hydrogenation in the batch reactor was carried out at 100 °C, 60 bar, with furfural in isopropanol (4.2:55.8 furfural:isopropanol volume ratio, 60 mL) for 2 h. S.A. Selishcheva et al. performed these experiments to find the most active and selective catalyst. The catalysts containing both copper and iron were very selective towards furfuryl alcohol (94–98%), while furfural conversion of more than 90% was achieved only on three samples: Cu_10_Fe_74_Al_16_, Cu_20_Fe_66_Al_14_, and Cu_30_Fe_57_Al_13_. The effective reaction rate constant was the highest for Cu_20_Fe_66_Al_14_. This catalyst was chosen for further experiments in the continuous flow reactor at 50 bar of hydrogen and varied temperatures of 100, 120, 140, and 160 °C. There is no explanation of such a difference in the reaction conditions of batch and continuous flow experiments. Temperatures up to 140 °C yielded only 22–33% of furfural conversion, which is much lower than values obtained in the batch reactor at 100 °C for 2 h. Raising the temperature to 160 °C allowed increasing conversion to 98%. In addition to furfuryl alcohol, 2-methylfuran was detected in the reaction products. One of the main conclusions of the present paper [42] is that the presence of highly dispersed copper particles determines the high activity and selectivity of Cu, Fe, and Al samples. However, comparison of the catalyst performance in batch and continuous flow modes was not the aim of the work. In any case, it is difficult to make any correlations between the two operation modes due to the difference in the reaction conditions performed. 

Y. Wang et al. [41,43] investigated the catalyst performance of monometallic Ni and bimetallic Ni-W catalysts supported on activated carbon (AC) in furfural hydrogenation to 2-methylfuran. The main studied reaction was hydrogenolysis with 2-metylfuran formation. Data concerning catalytic activity obtained in the batch reactor, as well as the reaction conditions, are present in Table 4. All batch experiments were performed in a 100 mL stainless steel autoclave with 300 mg of catalyst. Furfural hydrogenation experiments were performed for 5 h using a 0.35 M furfural feedstock in 60 mL of isopropanol (i-PrOH). Furfural conversion of 10% and FA selectivity of 99% was obtained over the monometallic Ni/AC catalyst at 200 °C in the absence of H_2_ [43]. Y. Wang et al. assumed that the reduction of furfural occurs through catalytic hydrogen transfer from i-PrOH to furfural, which was reported earlier only a few times [46,47]. Addition of hydrogen up to 30 bar resulted in an increase in conversion to 85%. Moreover, 2-methylfuran with a selectivity of 78% became the main product (Table 4), which is not common for Ni-based catalysts.

Usually, THFA is formed with a selectivity of 80–100%, probably due to the lower reaction temperature used (>140 °C) [48,49]. Much higher conversion of furfuryl (95%) was observed with the 5%Ni/AC catalyst with the temperature increase to 260 °C in the absence of hydrogen. At the same time, selectivity to MF and FA increases to 50% and increases to 20%, respectively, in comparison to the experiment performed at 200 °C in the absence of H_2_. The same trend in conversion change with varying reaction conditions is observed for W-doped Ni-based catalysts (Table 4) [43]. It was postulated in [46] that high MF selectivity in catalytic hydrogen transfer from i-PrOH to furfural can be promoted by a co-existing metal oxide phase, along with the metal phase. 

Further investigations were conducted using continuous flow reactors. Furfuryl hydrogenation in the case of Ni/AC and Ni-W/AC catalysts (110 mg) was performed using a Phoenix Flow Reactor in the absence of hydrogen [43]. The experiments over a monometallic Ni/AC catalyst were also conducted in H-Cube Pro equipment at an operation temperature of 150 °C due to equipment limits [41]. 

Stability test experiments were performed at optimized reaction conditions: 230 °C, pressure of 30 bar, and 0.1 M furfural in a Phoenix Flow Reactor [43]. The bimetallic 5%Ni–15%W/AC and 10%Ni–15%W/AC showed lower operation stability in comparison to monometallic 5%Ni/AC and 10%Ni/AC catalysts. Conversion dropped from 85 to 27% and from 99 to 11% for 5%Ni–15%W/AC and 10%Ni–15%W/AC, respectively, in 7 h, while for 5%Ni/AC, it decreased only to 80% in 10 h.

However, bimetallic catalysts are much more selective to MF in continuous flow experiments in comparison to the batch operation mode. The mean selectivity was 85 and 84% for the 5%Ni-15%W/AC and 10%Ni-15%W/AC catalysts, respectively, calculated over the 0.7–7.0 h period on the stream. The mass carbon balance was only 35–75% for the monometallic catalysts, while this value was 74–105% for the bimetallic catalysts, indicating that side products formation is suppressed in favor of MF formation. 

Y. Wang et al. [43] attributed high MF selectivity to the co-existence of NiO and Ni^0^ phases. According to XRD and XPS analysis, the NiO phase was present on the surface and in the bulk of the catalysts after performing hydrogenation of furfural in the continuous flow reactor, while this phase was present only on the surface of the samples after reaction in the batch reactor. This was caused by higher leaching of Ni in the batch, which was confirmed by ICP-MS data (Table 5). Additional leaching of W occurred from the 5%Ni-15%W/AC catalyst, and the higher W and Ni leaching was observed for the batch, which led to a decrease in MF selectivity (Table 5). It was postulated [43] that NiO and WO_x_ acted as the Lewis acid sites, promoting high MF selectivity in the batch and continuous flow for the 5%Ni/AC and 5%Ni–15%W/AC catalysts, respectively.

Thus, the 5%Ni/AC catalyst is less selective in the continuous flow reactor in comparison to the batch due to the lower concentration of Ni^0^ at the catalyst surface [43]. MF selectivity obtained over the 5%Ni–15%W/AC catalyst in the continuous flow is several times higher than that obtained in the batch due to the presence of NiO, Ni^0^, and WO_x_ species on the surface. 

The 5%Ni/AC catalyst was also tested in H-Cube Pro equipment [41]. The optimized temperature of 150 °C, pressure of 50 bar, and flow rate of 0.2 mL·min^−1^ were found to maintain the conversion of furfural as constant (~98–99%) over the Ni/AC catalyst. At the same time, MF selectivity remained quasi-constant at the level of ~20%, which was much lower in comparison to the 78% obtained in the batch mode. The main reason for such behavior can be the reaction temperature applied (150 vs. 200 °C). In addition, formation of MTHF and THFA in higher yields of 10–15% occurred. 

Based on the data presented, it can be concluded that the temperature of furfuryl hydrogenation plays a significant role in the formation of the desired product, whether it is furfuryl alcohol or 2-methylfuran, in the batch and continuous flow reactors. Batch mode is preferable for reaching practically full conversion of furfural with high 2-methylfuran selectivity (78%) with the 5%Ni/AC catalyst. However, the fresh catalyst should be added to the reactor for good catalytic performance since a high degree of Ni leaching was observed. High 2-methylfuran selectivity can be obtained also in the continuous flow mode with the 5%Ni–15%W/AC catalyst, whereas conversion of furfural will remain on the unsatisfied level. 

Later on [37], furfural hydrogenation to furfuryl alcohol was investigated over a Co/SiO_2_ catalyst in batch and continuous flow reactors. The catalyst was synthesized by the incipient wetness impregnation method, with following procedures of calcination and reduction in hydrogen flow. The hydrogenation of furfural in the batch reactor was carried out with 1 g of furfural and 9 g of ethanol over 50 mg of catalyst. The optimum reaction conditions for the Co/SiO_2_ catalyst were 150 °C, 20 bar, and 1 h, which allowed obtaining 100% conversion and 100% selectivity. Reuse of the catalyst was studied in four cycles, without any catalyst treatment after its recovering. After the fourth cycle, a significant drop in conversion (70%) and selectivity (90%) occurred due to sorption of furanic molecules [50] and formation of carbon deposits, which was confirmed by TEM measurements. To avoid this disadvantage, the reaction was performed in a continuous H-Cube ProTM flow reactor. The reactant amount was increased by five times (5 g of furfural and 45 g of ethanol) to fill the cartridge with catalyst (260 mg). The reaction conditions were 150 °C, 60 bar, and a flow rate of 0.3 mL·min^−1^. FAL conversion and FA selectivity remained constant for 180 min at the level of 100% and 90%, respectively. Used catalyst was investigated by ICP-MS and TEM. There was no leaching of cobalt or carbon deposition on the catalyst surface, meaning that the stability of the catalyst was higher under continuous flow processes due to the lower contact time between furanic molecules and the catalyst. Moreover, higher space–time yields were obtained when performing furfural hydrogenation in the continuous flow reactor than in the batch one (16.6 g·L^−1^·h^−1^ for 150 °C and 60 bar vs. 13.2 g·L^−1^·h^−1^ for 150 °C and 20 bar). According to the results [37], it can be concluded that continuous flow mode is preferential for furfural hydrogenation since it is possible to avoid formation of carbonaceous species on the catalyst surface, which led to deactivation of the catalyst. 

Since Cu-containing materials are active and selective catalysts in furfural hydrogenation, they can be used in HMF hydrogenation, which was demonstrated in [51]. A mesoporous Cu-Al_2_O_3_ catalyst was synthesized by grounding metal salts and NH_4_HCO_3_ in a mortar, with following calcination of the resulting paste and its reduction in a hydrogen flow. The batch experiments were performed in a glass-lined batch reactor with 0.25 g of catalyst and 0.5 g HMF in ethanol as a solvent. The effect of hydrogen pressure and the reaction temperature on the catalyst performance was investigated (Table 6). 

As it is seen, high HMF conversion (95%) was achieved in the temperature range of 60–100 °C at H_2_ pressure of 50 bar. A decrease in hydrogen pressure to 10 bar at 70 °C led to a conversion drop to 24.5%. Selectivity to BHMF remained at a high level (98%), independent of the reaction conditions.

The recyclability of the mesoporous Cu-containing catalyst was studied at 70 °C and 50 bar. The BHMF yield and HMF conversion decreased from 98.6 and 99.1% in the first run to 74.2 and 76.6%, respectively, in the third run [51]. 

The further catalytic tests were performed in the continuous flow mode using a stainless steel reactor (12 mm inner diameter and 400 mm length) at a hydrogen pressure of 50 bar, temperature of 100 °C, and weight hourly space velocity of 0.2 h^−1^ to imitate the residence time in the batch experiment, which was performed for 3.5 h. At the beginning of the experiment, the BHMF yield was 98%, which dropped to 92% after 68 h and remained constant for another 32 h. In the case of the batch reactor, the BHMF yield achieved 97.8% at 100 °C and 50 bar. Thus, mesoporous Cu/Al_2_O_3_ catalyst shows similar activity and selectivity in batch and continuous flow reactors. However, the continuous flow mode is preferable for long-term measurements since the catalyst can work stably, without a significant loss in selectivity for at least 100 h, and does not require a reloading procedure, whereas the catalyst deactivates in the third run after 10.5 h of performing the reaction in the batch reactor.

### 3.2. Hydrogenation of Levulinic Acid to γ-Valerolactone

**Scheme 2:**
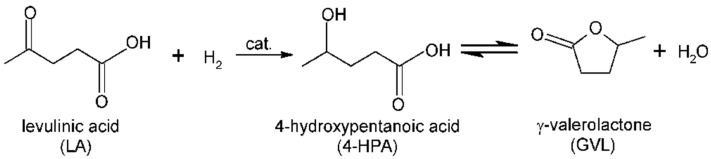
Hydrogenation of levulinic acid.

Except for furan molecules, another promising chemical produced from lignocellulosic biomass is levulinic acid [52], the starting material for the production of γ-valerolactone (GVL) [53,54]. GVL can be widely used as a fuel additive or green solvent [55,56]. GVL is usually synthesized by levulinic acid hydrogenation; besides GVL, the intermediate 4-hydroxypentanoic acid (4-HPA) can be also detected (Scheme 2). 

H.C. Genuino et al. [57] studied the catalytic performance of the 1 wt.% Ru/ZrO_2_ catalyst in LA hydrogenation to GVL in batch and continuous flow setups and the effect of formic acid in the reaction feed on the GVL yield. Formic acid is an inevitable impurity in levulinic acid, being the byproduct of LA formation from sugar sources. Batch studies experiments were performed in a 50 mL Parr batch autoclave, while continuous flow reactions were carried out in a stainless-steel reactor (inner diameter of 3.6 mm and length of 5 cm). 

The batch autoclave reactor was loaded with the substrate (0.0258 mol LA), catalyst (0.75 g), and dioxane (30 mL). The experiments were performed at 150 °C and a hydrogen pressure of 50 bar. During the reaction, samples were taken after 30, 60, 120, 180, and 300 min of the experiment. In the absence of formic acid, the combined GVL+4-HPA yield was 100% in 30 min of the experiment. A continuous decrease to 90% was observed in 300 min. 

H.C. Genuino et al. [57] studied the effect of the formic acid concentration on the catalyst performance by varying the molar HCOOH:LA ratio in the range of 1:20–1:1. Regardless of the formic acid concentration, the initial activity for LA hydrogenation was zero. A certain induction period for catalyst activity was observed. The higher the molar HCOOH:LA ratio, the longer the induction period was (Table 7).

H.C. Genuino et al. [57] claimed that, at first, HCOOH converted to CO_2_, H_2_O, and CO, which was confirmed by gas analysis of the gas products of benchmark experiments of formic acid conversion at 150 °C, without LA and H_2_, over Ru/ZrO_2_. LA hydrogenation occurred only after HCOOH conversion. The recyclability of the Ru/ZrO_2_ catalyst was studied in LA hydrogenation with a molar HCOOH:LA ratio of 8 after 300 min of the experiment in six runs. Spent catalyst was washed with acetone and dried at 60 °C and reused afterwards in LA hydrogenation. The GVL yield of ~95% remained unchanged for six cycles. This means that the catalyst inhibition with HCOOH was fully reversible. 

Impurity-free long-term experiments (190 h) were performed in a continuous flow reactor at 150 °C and an H_2_ pressure of 50 bar. The catalyst amount was 100 mg. The initial in situ activation of the catalyst was observed. This was explained by the redispersion of ruthenium (detected by TEM measurements) at the beginning of the long-term experiment [57]. LA conversion dropped from 100 to ~50% in 190 h because of coke formation on the catalyst surface that was confirmed by TG analysis.

The effect of formic acid on catalyst performance was investigated by switching between levulinic acid (10 wt.%) solution with solvent to solution additionally containing 5 wt.% HCOOH. According to calculations, the LA concentration in the batch experiment was 8.8 wt.%, and the formic acid concentrations were in the range of 0.17–3.37 wt.%. The values are quite close to those used in continuous flow experiments. Addition of HCOOH in 30 h of the experiment led to a severe decrease in formation of target GVL+4-HPA, from ~92% to zero yields in another 10 h, meaning complete loss of the catalyst activity. Switching back to pure levulinic acid solution (total of 55 h of the experiment) resulted in regaining catalytic activity in the values of GVL yields (~85%) in view of the catalyst deactivation in the benchmark test. Ten hours were needed to regain catalytic activity due to adsorption of HCOOH on the Ru catalyst surface, followed by its conversion to CO, CO_2_, and H_2_ [58,59]. Thus, reactivation of the catalysts was possible by removing formic acid from the reactants feed. The obtained data are in agreement with results obtained for LA hydrogenation in the presence of formic acid in the autoclave. 

Thus, H.C. Genuino et al. [57] showed that formic acid, which is an inevitable byproduct of levulinic acid formation from sugar sources, deactivates the catalyst. However, removing formic acid from the reactant feed regained catalytic activity. Combined GVL+4-HPA yields were at the same level for both operation modes. 

G. Grillo et al. [60] investigated hydrogenation of levulinic acid to GVL under microwave (MW) irradiation in batch or continuous flow processes over Ru/AC and Ru/TiO_2_ catalysts. It was suggested [60] that using microwave irradiation can improve conversion of levulinic acid in a shorter time. MW-assisted batch experiments were performed in a high-pressure MW reactor at 150 °C, H_2_ pressure of 5–10 bar, and catalyst/LA ratio of 0.7–0.8 mg_cat_·mg_LA_^−1^. Full conversion of levulinic acid and GVL selectivity of 100% were obtained at a hydrogen pressure of 7 bar in 2 min over 3 wt% Ru/AC, in spite of partially agglomerated Ru particles. The carbon balance was 94%. On the other hand, the higher hydrogen pressure of 10 bar and higher catalyst/LA ratio of 0.8 were necessary to obtain complete LA conversion with a carbon balance of 98% over 3 wt% Ru/TiO_2_. The better catalyst performance of the Ru/AC catalyst in comparison to the Ru/TiO_2_ catalyst was explained by the larger surface area of activated carbon due to its porosity. TEM measurements of spent and as-synthesized Ru/AC catalyst showed that the particle size distribution remained unchanged after levulinic acid hydrogenation, meaning the absence of ruthenium coalescence or leaching during the reaction. 

MW-assisted continuous flow experiments were performed in a multiphase multimodal reactor at 150 °C and a hydrogen pressure of 10 bar to study the catalysts stability. The catalyst/LA ratios of 0.35 and 0.4 mg_cat_·mg_LA_^−1^ were used in the case of Ru/AC and Ru/TiO_2_ catalysts, respectively. The complete conversion of levulinic acid was achieved in 8 min for both catalysts and remained unchanged for 30 min. However, the carbon balance of 35 (Ru/AC) and 51% (Ru/TiO_2_) was much lower in comparison to batch experiments. G. Grillo et al. [60] explained this low carbon balance by the formation of carbonaceous particles on the catalyst surface, which was detected by EDS analysis. According to ICP-OES analysis of the spent catalysts, a metal leaching of 9.8% occurred in the case of the Ru/AC catalyst, while Ru/TiO_2_ showed high stability towards metal leaching due to the strong interaction between the metal and support. 

It was claimed [60] that both ruthenium catalysts are strong prospective systems from the industrial point of view due to their efficiency in continuous flow experiments for obtaining complete conversion of levulinic acid and selectivity towards GVL. However, it should be noted that stability experiments in the continuous flow setup were performed only for 30 min, which is not enough for industrial use. In addition, the metal leaching from the catalyst, as well as the formation of carbon deposits on the catalyst surface, already occurred during these 30 min. Increasing reaction time will lead to an increase in metal leaching and the number of carbonaceous particles blocking the active catalyst sites. Thus, further experiments on microwave-assisted hydrogenation of levulinic acid in a continuous flow reactor should be carried out. 

Unlike other supports, ZrO_2_ itself can be used as the catalyst in the catalytic transfer hydrogenation (CTH) of methyl or ethyl levulinate to GVL [61] due to bifunctional properties of ZrO_2_ surface sites [62]. CTH reactions use an indirect hydrogen source, such as alcohol molecules (H-donors). Levulinate derivative is obtained by the esterification reaction of LA with alcohol or directly from cellulose. They are beneficial in comparison to LA for industrial use due to their lower boiling point and free acid parameters [63]. 

T. Tabanelli et al. [63] compared the performance of a ZrO_2_ catalyst in the transfer hydrogenation of ethyl levulinate (EL) in both continuous gas-flow and batch reactors. Batch experiments were performed in a stainless-steel autoclave with 0.3 g of catalyst at 250 °C, an N_2_ pressure of 10 bar, and 10 wt.% of ethyl levulinate in i-PrOH for 8 h. The GVL yield was 27%, with EL conversion of 31% due to the high ability of i-PrOH for releasing hydrogen [64]. Increasing the reaction time to 24 h allowed increasing the EL conversion and GVL yield to 79 and 64%, respectively. Recycling tests were repeated four times. GVL selectivity remained practically unchanged at ~80%, while the activity dropped significantly from ~77 to ~30%, meaning that the GVL yield also decreased with repeating experiments. However, heat treatment of the spent catalyst at 400 °C for 2 h resulted in the return of the catalyst activity to the initial level, indicating the formation of carbon deposits on the catalyst surface in the course of the reaction, which can be removed by proper heating.

Continuous flow experiments were performed in a tubular glass reactor (length of 450 mm and inner diameter of 19 mm) at 250 °C and a molar ratio of EL:i-PrOH of 1:10 with a residence time of 1 s [63]. The weight percentage of EL was higher in comparison to the batch experiment (20 vs. 10 wt.%). Complete and stable conversion of EL was obtained, with a GVL yield of ~50% in 7 h of the experiment. However, the carbon balance did not reach 100%, meaning substrate overreduction to light compounds, which were not detected. The long-term stability test was performed for more than 750 min of time-on-stream. The activity of ZrO_2_ started to decrease in 600 min: the EL conversion and GVL yield was less than 90% and 30%, respectively. Therefore, T. Tabanelli et al. [63] performed regeneration of the catalyst by stopping the reagent gas mixture and purging the catalyst with air at 400 °C for 2 h. After this procedure, the EL conversion and GVL yield returned to the initial levels. According to TGA, the loss in activity was associated with formation of carbon deposits. 

Thus, T. Tabanelli et al. [63] showed that a tetragonal ZrO_2_ catalyst can be active and selective in a CTH reaction under both batch and continuous gas flow conditions. The catalyst regeneration can be performed in situ in a continuous flow reactor, while the spent catalyst should be taken out from the batch reactor and regenerated in another setup that needs additional time. 

### 3.3. Hydrogenation of Cinnamaldehyde 

Selective hydrogenation of cinnamaldehyde to cinnamyl alcohol has attracted a lot of attention due to wide use of the latter in perfumes and flavorants [65]. L.J. Durndell et al. [66] studied Pt-catalyzed cinnamaldehyde hydrogenation in a Parr stainless steel, stirred, batch autoclave and in a commercial continuous Uniqsis FlowSyn reactor. The catalyst in the continuous flow reactor was located in an OMNIFIT^®^ glass column (inner diameter of 10 mm and length of 100 mm). The reaction was performed at the same reaction conditions: 8.4 mmol cinnamaldehyde, 90 °C, H_2_ pressure of 5 bar, 200 mg of catalyst. The stability and productivity of cinnamyl alcohol was studied in both reactors for 7 h. It was shown that TOF was higher in the continuous flow mode than that in the batch one (1300 h^−1^ vs. 600–850 h^−1^). Such a difference could be caused by a fast catalyst deactivation in the batch reactor due to catalyst poisoning by the adsorbed product. In the case of the flow reactor, the possible poisons were removed from the catalyst surface by a feed flow. Formation of cinnamyl alcohol with a selectivity of 60%, as well as 3-phenylpropionaldehyde, ethylbenzene, and 3-phenylpropan-1-ol, was observed in flow experiments. The catalyst worked stably for 7 h without changing selectivities. 

The main reaction in the batch reactor was hydrogenation of the C=C bond of cinnamaldehyde to 3-phenylpropionaldehyde, which selectively decreased from 50 to ~42% with time [66]. The high selectivity of ~45% towards 3-phenylpropan-1-ol was observed at the beginning of the reaction, meaning the direct hydrogenation of the formed cinnamyl alcohol to this product. Catalyst deactivation (TOF decrease from 850 to 600 h^−1^) was accompanied by an increase of the cinnamyl alcohol selectivity from 8 to 45% and a decrease of the 3-phenylpropan-1-ol selectivity from 45 to 10%, indicating that different Pt active sites were responsible for hydrogenation of C=O and C=C bonds [67]. Comparison of the obtained products in the continuous flow and batch reactors revealed that the main catalyst poison was ethylbenzene. In the case of the fixed-bed reactor, ethylbenzene was carried away from the catalyst surface by the flow, whereas it remained adsorbed on the catalyst surface in the batch reactor [66]. The productivity of the cinnamyl alcohol collected for 7 h was ~19 and ~58 mmol·g_cat_^−1^ in the batch and continuous flow reactor, respectively. Thus, L.J. Durndell et al. showed that performing Pt-catalyzed cinnamaldehyde hydrogenation was preferable in the continuous flow reactor since it was possible to remove ethylbenzene, a catalyst poison, fast from the catalyst surface. 

## 4. Hydrogenation of Unsaturated Carbon–Carbon Bonds

### 4.1. Semihydrogenation of Carbon Triple Bonds

Obtaining (Z)-alkenes from alkynes by their hydrogenation is an important reaction for constructing the precursor of bioactive or industrially valuable functional materials [68,69]. Yamada et al. [69] developed a Pd catalyst supported on silicon carbide (3%Pd/SiC), which provides high chemical stability. Chemoselective hydrogenation of alkynes was investigated over this catalyst at an ambient H_2_ pressure and temperature (25 °C) in methanol (0.25 M) under batch and continuous flow conditions. 

The first series of experiments for semihydrogenation of different alkynes were performed in a batch reactor over 8.8 mg of 3%Pd/SiC in the presence of DETA as additive (1.5 equivalents towards substrate) for 2–4 h to study the efficiency of this approach in the formation of Z-alkene. It was found that disubstituted alkynes were hydrogenated with high conversions (99%) and excellent selectivities towards Z-alkynes (98%). Monosubstituted alkynes, such as 2-ethynyl-6-methoxynaphthalene and N-Cbz-protected 4-ethynyl aniline, were transformed to corresponding alkenes with a selectivity of 96 and 90%, respectively. However, a quite low isolated yield of 4-aminostyrene (67%) was obtained in 2 h of the reaction. Yamada et al. [69] assumed that this happened due to the competitive coordination of the aromatic amino group of the substrate and product to Pd species instead of DETA. Therefore, to avoid competitive coordination of amino substrates to Pd species, another catalyst with immobilized DETA on 3%Pd/SiC (3%Pd(DETA)/SiC) was developed. Using such a catalyst resulted in an increase in the isolated yield of 4-aminostyrene to 92% in 1 h of the reaction. Thus, using immobilized DETA on 3%Pd/SiC allowed increasing the yield of 4-aminostyrene in a shorter reaction time.

Semihydrogenation of alkynes in a continuous flow reactor packed with 50 mg of catalyst (3%Pd/SiC) was carried out by pumping a solution of alkyne (0.5 mmol) and DETA (3.0 equivalents) in MeOH (0.25 M) into the catalyst cartridge. There is no explanation for using a higher amount of DETA in flow experiments in the paper [69]. It was found that disubstituted and monosubstituted alkyne can be selectively hydrogenated to Z-alkynes with excellent-to-high yields (80–95%) with a residence time of 1–2 min. Long-term stability experiments in a continuous flow reactor was performed using 2,5-dimethyl-3-hexyne-2,5-diol as a substrate for 24 h. The Z-alkene yield was 95% after the first 3 h of the experiment over the 3% Pd/SiC catalyst. However, after that, it started to drop and reached 65% in 24 h. It was postulated that the catalyst deactivation could be associated with excess amounts of DETA, which strongly coordinates with 3% Pd/SiC, during the flow reaction, resulting in blocking active sites of the catalyst. Using 3% Pd(DETA)/SiC as the catalyst allowed obtaining 90% of the desired Z-alkene and remained unchanged for 24 h. Thus, Yamada et al. [69] showed that when performing semihydrogenation of alkenes, the possible coordination of functional groups of additives or substrates with active catalyst species should be taken into account, both in batch and continuous flow reactors. The reaction conditions should be chosen in such a way that Z-alkene will be close to 90–100%. 

N. Numwong et al. [70] investigated partial hydrogenation of polyunsaturated FAME over a Pd/C catalyst. FAME can be used as an alternative fuel in comparison to petroleum-based fuels [71,72]. Oxidative stability and cold-flow properties are important features of FAME. The lower the unsaturated fatty acid concentration in biodiesel, the higher the oxidative stability is. In contrast, biodiesel with a high concentration of saturated fatty acids has worse cold-flow properties [73,74]. A balance between these parameters is needed to obtain biodiesel with suitable characteristics. 

Partial hydrogenation of FAME was performed under batch and continuous flow conditions [70]. Batch experiments were carried out in a stainless steel semi-batch reactor at 120 °C and a hydrogen pressure of 4 bar over 1.5 g of the catalyst. N. Numwong et al. [70] monitored the transformation of C18:0, C18:1, C18:2, and C18:3 fatty acids in the course of the reaction (Table 8). 

Despite the fact that the reaction was performed for 4 h, better fuel properties were obtained after 1.5 h of the experiment due to the full conversion of polyunsaturated FAMEs (C18:2 and C18:3) to monounsaturated C18:1, with a minimal formation of saturated C18:0. The oxidative stability of the obtained biodiesel was 32.5 h, which is much higher in comparison to that of untreated biodiesel feed (1.49 h). 

The partial hydrogenation of polyunsaturated FAMEs in a continuous flow reactor was conducted at 120 °C, an H_2_ pressure of 4 bar, and varied feed flow rates (20–180 g·h^−1^). The conversion increased from 44.2% to 96.2% with the increase in contact time from 0.0011 (180 g·h^−1^) to 0.01 h (20 g·h^−1^) using 0.2 g of catalyst, which was comparable to the conversion obtained in the batch reactor (58.6–98.3%) with a much higher amount of the catalyst (1.5 g). This means that a higher reaction rate per unit amount of catalyst was observed in the continuous flow reactor. However, the higher degree of C18:1 hydrogenation to C18:0 was obtained with increasing contact time.

N. Numwong et al. [70] made a comparison of the catalyst performance in batch and continuous flow reactors at 120 °C and 4 bar of H_2_ by comparison of the FAME composition of the biodiesel product at the same conversion. At low conversion < 78%, similar selectivity towards C18:1 was obtained in both reactors. However, high conversion of 94.5% resulted in a high degree of hydrogenation of C18:1 and formation of a high amount of saturated C18:0 in the continuous flow reactor, which negatively affects the cloud and pour points. Thus, it was shown that performing partial hydrogenation of polyunsaturated FAMEs with high conversion of C18:2 and C18:3 and high selectivity to C18:1 in a batch reactor is preferable. However, a continuous flow reactor is better to use at a lower conversion due to the lower residence time in comparison to a batch reactor.

It can be concluded that N. Numwong et al. [70] discovered the benefits of performing partial hydrogenation of FAMEs in a batch reactor to obtain a biodiesel product with desired properties. Moreover, it was shown that a continuous flow reactor also can be used for this by carrying out partial hydrogenation of FAME at the middle conversions. 

### 4.2. Selective Hydrogenation of Double Carbon Bonds

**Figure 2:**
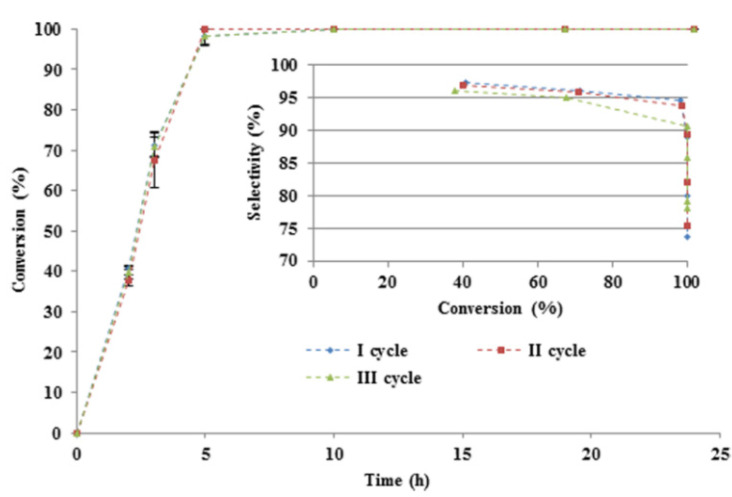
Conversion of limonene and selectivity towards (+)-p-1-menthene over Pt/C in three cycles in the batch reactor. Reprinted (adapted) with permission from Rubulotta, G.; Luska, K. L.; Urbina-Blanco, C. A.; Eifert, T.; Palkovits, R.; Quadrelli, E.A.; Thieuleux, C.; Leitner, W. Highly Selective Hydrogenation of R-(+)-Limonene to (+)-p-1-Menthene in Batch and Continuous Flow Reactors. ACS Sustainable Chem. Eng., 2017, 5, 3762−3767. Copyright 2017 American Chemical Society.

(+)-P-1-menthene can be used as a starting material for the synthesis of adhesives, coatings, food, steroids, antimalarial agent, and menthol [75,76]. One of the possible preparation methods of (+)-p-1-menthene is selective hydrogenation of limonene [77]. The partial hydrogenation was carried out over the Pt/C catalyst under batch and continuous flow conditions. Batch experiments were performed at an H_2_ pressure of 3 bar, 30 °C, a catalyst with 1.5 μmole of metal, and 1 mL neat limonene in a stainless steel high pressure reactor, equipped with a glass insert. A high (+)-p-1-menthene selectivity of 95% was obtained after 2 h, while it dropped to 83% at the complete conversion of limonene due to the conversion of the target product to p-menthane. To evaluate the stability of the catalyst, recycling tests were performed at the same reaction conditions after 24 h of the experiment. Before each run, the catalyst was separated from the reaction mixture and dried under argon and a vacuum. The catalyst remained stable, without a loss in activity and selectivity in the third cycle (Figure 2). 

ICP-MS was performed to determine Pt leaching. In supernatant, 0.0016% of Pt was detected, meaning a high stability of the catalyst in these reaction conditions. Therefore, limonene hydrogenation was further performed under continuous flow conditions using an H-Cube Pro reactor at an H_2_ pressure of 20 bar, 0.3 M solution of limonene in EtOH, a substrate flow of 2.5 mL·min^−1^, and 260 mg of 5%Pt/C catalyst. Full limonene conversion remained unchanged for 4 h, with a little drop to 96% in the next 3 h. Selectivity towards (+)-p-1-menthene was quite stable, at the level of 87–92%. Thus, G. Rubulotta et al. [77] showed that Pt/C catalyst is a prospective material for partial hydrogenation of limonene to (+)-p-1-menthene. 

It should be noted that this work does not really compare the catalyst performance under batch and continuous flow conditions since quite different reaction conditions were used for both modes: 3 mg and 260 mg of the catalyst, respectively, or 3 and 20 bar of hydrogen, respectively. The focus of the work was on finding the optimal conditions to achieve full conversion of limonene with a high selectivity to the target product for batch and continuous flow modes. Furthermore, the amount of catalyst in the batch reactor was very low, which makes it difficult to evaluate the possibility of scaling up this process

## 5. Conclusions

In this review article, we analyzed the data devoted to the selective hydrogenation of functional groups (nitro and carbonyl groups and unsaturated carbon bonds) in organic compounds. We considered only publications that investigated the catalyst performance under batch and continuous flow conditions that allowed finding benefits and drawbacks of both regimes. Authors [77] used the batch reactor to find optimal reaction conditions, while the continuous flow reactor was used to estimate catalyst stability in long-term experiments. In other publications [12,22,37,41,66], it was only shown that both regimes can be applied for selective hydrogenation of functional groups, without any conclusions about which reactor should be used since completely different reaction conditions were applied. Some works [24,25,60] consider quite specific batch and continuous flow reactors, whose industrial use is in doubt. However, very few [13,43,57,70] papers really compare catalytic activity and selectivity in batch and continuous flow reactors. 

It was shown [14] that performing selective hydrogenation of o-chloronitrobenzene over the Au/TiO_2_ catalyst in a continuous flow reactor is preferable in comparison to batch reactor due to reducing working time with the increase in productivity to target chloroaniline. Decrease in working time is caused by exclusion of two steps (extraction of product/recharge with reactant, catalyst filtration/washing/reloading) from the process. 

It was found [21,22] that different Co catalysts are promising catalysts in selective hydrogenation of iodonitrobenzene. However, the batch reactor should be designed especially for Co catalyst to exclude the influence of noble metals residues, whose presence result in formation of undesired aniline [23]. The continuous flow mode is preferable for obtaining high 4-iodoaniline yield due to the possibility to choose the optimum residence time.

Furfural hydrogenation was investigated over Ni-containing catalysts [43]. It was shown that the batch reactor is preferable to obtain practically full conversion of furfural with high 2-methylfuran selectivity (78%) over the 5%Ni/AC catalyst. The 5%Ni–15%W/AC catalyst revealed high selectivity towards 2-methylfuran in continuous flow mode, but with low furfural conversion, meaning that optimal reaction conditions should be found to perform hydrogenation in the flow mode with high selectivity and activity. 

On the other hand, continuous flow mode is preferential for hydrogenation of carbonyl groups over a different catalyst since it is possible to remove hydrocarbon poisons from the catalyst surface by gas flow or regenerate the catalyst by removing carbonaceous species in situ in continuous flow reactors. However, the spent catalyst after the batch experiment should be taken out from the reactor and regenerated in another setup that needs additional operation time. 

Thus, the undoubted benefit of continuous flow reactors is an ease of maintenance, which lies in the fact that the catalyst does not need to be separated from the reaction products and subjected to washing for subsequent use. On the other hand, the productivity, selectivity, and stability of the catalyst can be beneficial both in the batch and flow reactors, depending on the reaction parameters.

In our opinion, little attention is paid to the fact that the flow reactor allows the activation of the catalyst under optimal conditions. It is especially important to the catalysts, which can be oxidized on contact with air after their activation in hydrogen. The batch reactor does not allow reducing the catalyst in situ. This means that usage of the batch reactor is limited to the catalysts that are air-stable in their reduced state since the catalyst is reduced in the separate setup and transferred afterwards to the reactor. In the case of the flow reactor, the catalyst can be activated by hydrogen in situ, followed by feeding the reaction mixture without air contact.

## Figures and Tables

**Table 1:** Catalytic activity in batch liquid- and continuous gas-phase operations depending on reaction conditions over different catalysts.

Catalyst	Operation Mode	Solvent	Substrate	P_H2_, atm	T, °C	S_CAN_/S_AN_/S_NB_^b^	r ^c^	Reference
Pd/C	Batch liquid	Ethanol	o-CNB	5	150	79/20/1	2290	[13]
Pd/C		Ethanol	o-CNB	10	150	81/18/1	2650	[13]
Pd/C		Ethanol	o-CNB	12	150	86/13/1	2910	[13]
Au/TiO_2_		Ethanol	o-CNB	5	150	100/0/0	10	[13]
Au/TiO_2_		Ethanol	o-CNB	12	150	100/0/0	167	[13]
Au/Mo_2_N		n.m. ^a^	p-CNB	11	150	100/0/0	-	[12]
Au/Mo_2_N	Continuous gas	Ethanol	o-CNB	1	220	100/0/0	42	[12]
Au/TiO_2_		Ethanol	o-CNB	1	150	100/0/0	12	[13]
Au/TiO_2_		Ethanol	p-CNB	1	150	100/0/0	12	[13]
Au/Al_2_O_3_		Ethanol	p-CNB	1	220	100/0/0	12	[12]
Pd/Mo_2_N		Ethanol	p-CNB	1	220	~55/5/45	66	[12]

^a^ not mentioned. ^b^ S_CAN_, S_AN_, S_NB_—selectivity to chloroaniline, aniline, and nitrobenzene, respectively. ^c^ mol_CNB_/(mol_met_*h).

**Table 2:** Catalytic activity in 1-iodo-4-nitrobenzene hydrogenation over Raney Co and Co EnCatTM in batch experiments.

Reaction Conditions	110 °C, 20 Bar of H_2_, 10 mL of THF, 80 min, Raney Co			100 °C, 85 Bar of H_2_, 10 mL of THF:H_2_O (95:5), 40 min, Co EnCat^TM^		
C Substrate, mol/L	INB Conversion, %	IAN yield, %	Yield (Aniline), %	INB Conversion, %	IAN Yield, %	Yield (Aniline), %
0.05	82	48	<2	91.6	70.4	3.1
0.1	77	50	<2	90.9	71.8	3.1
0.2 *	81	42	<2	79.0	60.7	3.3

* the reaction time was 100 min.

**Table 3:** Reaction conditions of 4-nitrophenol hydrogenation in the batch experiments.

Catalyst	Amount, mg	Temperature, °C	4-Nitrophenol Amount		NaBH_4_ Amount	Reference
			mL	M	mL	
Cu_x_O/Cu/NC	0.075–0.125	25	0.027	0.01	0.27	[25]
cAg-NF	0.450 (Ag-NF)		0.07		7	[24]

**Table 4:** Furfural conversion and product yields after selective hydrogenation in batch reactor over different catalysts.

Catalyst	T, °C	P(H_2_), bar	Conversion, %	Yield, %		Selectivity MF, %
				FA	MF	
5%Ni/AC	200	0	10	10	0	0
	200	30	85	6	66	78
	260	0	95	20	50	53
5%Ni-15%W/AC	200	30	50	32	15	30
	260	0	83	16	6	7
10%Ni-15%W/AC	200	30	51	8	20	39
	260	0	83	25	6	7

**Table 5:** Ni and W content as determined by XPS and ICP-MS analysis in fresh and used mono- and bimetallic Ni catalysts.

Catalyst	XPS, wt.%			ICP-MS, wt.%		Selectivity MF, %
	NiO	Ni^0^	W	Ni	W	
5% Ni/AC, fresh	0.5	0.4	-	4.1	-	-
5%Ni/AC, used (batch, 200 °C, 30 bar H_2_)	0.3	0.2	-	2.3	-	78
5%Ni/AC, used (batch, 260 °C, no H_2_)	0.6	0.6	-	2.4	-	53
5%Ni/AC, used (continuous flow, 260 °C, 30 bar, no H_2_)	0.7	0.2	-	3.9	-	40 ^b^
5%Ni–15%W/AC, fresh	0.5	0.1	17.4	4.3	0.1 ^a^	-
5%Ni–15%W/AC, used (batch, 260 °C, no H_2_)	0.2	0.0	5.6	2.6	0.1 ^a^	7
5%Ni–15%W/AC, used (continuous flow, 260 °C, 30 bar, no H_2_)	0.7	0.1	15.4	3.2	0.1 ^a^	85 ^b^

^a^ Not quantitative due to insoluble W-containing complex formation; ^b^ Mean selectivity calculated over the 0.7–7.0 period on the stream.

**Table 6:** Effect of reaction conditions on HMF conversion and BHMF selectivity in the batch experiments.

H_2_ Pressure, bar	Temperature, °C	HMF Conversion, %	BHMF Selectivity, %
50	60	95.0	98.0
50	70	99.1	99.5
50	100	98.7	99.1
30	70	97.5	98.1
20	70	97.0	97.9
10	70	24.5	98.0

**Table 7:** Combined GVL and 4-HPA yields depending on the molar HCOOH:LA ratio in batch experiments.

HCOOH:LA Ratio	Induction Period, Minutes	GVL+4-HPA Yield, %			
		60 min	120 min	180 min	300 min
1:20	60	5	100	100	-
1:10	120	~2	5	100	95
1:8	180	~2	~2	12	100
1:5	-	~2	~2	~2	-
1:1	-	0	0	0	2

**Table 8:** C18 FAMEs composition (%) of biodiesel after partial hydrogenation in a batch reactor.

Reaction Time, h	C18:2	t-C18:1	c-C18:1	C18:0
0	5	0	30.5	6
0.5	2.5	5	27.5	7
1	1	12.5	21	7
1.5	0	18	14	9.5
2	0	19.5	12	12
3	0	19	10	14
4	0	9	16	17.5

## Data Availability

All relevant data are within the paper.
